# Epigenetic and transcriptome responsiveness to ER modulation by tissue selective estrogen complexes in breast epithelial and breast cancer cells

**DOI:** 10.1371/journal.pone.0271725

**Published:** 2022-07-21

**Authors:** Terri L. Messier, Joseph R. Boyd, Jonathan A. R. Gordon, Coralee E. Tye, Natalie A. Page, Rabail H. Toor, Sayyed K. Zaidi, Barry S. Komm, Seth Frietze, Janet L. Stein, Jane B. Lian, Gary S. Stein

**Affiliations:** 1 Department of Biochemistry, University of Vermont Larner College of Medicine, Burlington, VT, United States of America; 2 University of Vermont Cancer Center, University of Vermont Larner College of Medicine, Burlington, VT, United States of America; 3 Komm Pharma Consulting LLC, San Francisco, CA, United States of America; 4 Department of Biomedical and Health Sciences, University of Vermont, Burlington, VT, United States of America; 5 Department of Surgery, University of Vermont, Burlington, VT, United States of America; Roswell Park Cancer Institute, UNITED STATES

## Abstract

Selective estrogen receptor modulators (SERMs), including the SERM/SERD bazedoxifene (BZA), are used to treat postmenopausal osteoporosis and may reduce breast cancer (BCa) risk. One of the most persistent unresolved questions regarding menopausal hormone therapy is compromised control of proliferation and phenotype because of short- or long-term administration of mixed-function estrogen receptor (ER) ligands. To gain insight into epigenetic effectors of the transcriptomes of hormone and BZA-treated BCa cells, we evaluated a panel of histone modifications. The impact of short-term hormone treatment and BZA on gene expression and genome-wide epigenetic profiles was examined in ERα^neg^ mammary epithelial cells (MCF10A) and ERα^+^ luminal breast cancer cells (MCF7). We tested individual components and combinations of 17β-estradiol (E2), estrogen compounds (EC10) and BZA. RNA-seq for gene expression and ChIP-seq for active (H3K4me3, H3K4ac, H3K27ac) and repressive (H3K27me3) histone modifications were performed. Our results show that the combination of BZA with E2 or EC10 reduces estrogen-mediated patterns of histone modifications and gene expression in MCF-7^ERα+^ cells. In contrast, BZA has minimal effects on these parameters in MCF10A mammary epithelial cells. BZA-induced changes in histone modifications in MCF7 cells are characterized by altered H3K4ac patterns, with changes at distal enhancers of ERα-target genes and at promoters of non-ERα bound proliferation-related genes. Notably, the ERα target gene *GREB1* is the most sensitive to BZA treatment. Our findings provide direct mechanistic-based evidence that BZA induces epigenetic changes in E2 and EC10 mediated control of ERα regulatory programs to target distinctive proliferation gene pathways that restrain the potential for breast cancer development.

## Introduction

Menopausal hormone therapy (MHT) has traditionally been the most frequently used treatment for post-menopausal symptoms. However, controversy regarding its use was raised by findings from the Women’s Health Initiative study [1], which was stopped prematurely due to evidence of increased risk of heart attack, stroke, and breast cancer. These findings led to a decline in the use of MHT to manage menopausal symptoms [2]. Consequently, considerable effort has been directed toward development of new approaches to MHT, with the goal of minimizing cancer risk while providing positive physiological effects on the cardiovascular system, bone homeostasis, and the central nervous system.

Development of selective estrogen receptor modulators (SERMs, e.g., tamoxifen, raloxifen) provides a clinical strategy for a therapeutic response with specific tissue-protective benefits [3–7]. However, SERMs alone do not have maximal physiological effects. Here we examined bazedoxifene (BZA), which has mixed properties dependent on the tissue. It functions as a partial agonist in bone where it has beneficial effects for treating osteoporosis, but is neutral in uterine epithelium. BZA also functions as an antagonist by inducing degradation of ERα in breast cancer cells [8]. Further, there is precedence from previous studies [9–11] for the advantage of BZA in combination with estrogenic compounds. The combination of one or more estrogens with a SERM or SERD (selective estrogen receptor degrader) results in a tissue selective estrogen complex (TSEC) with the desired beneficial estrogen receptor agonist activity balanced with the antagonistic activity in breast tissues, thereby minimizing the negative effects of estrogen therapy alone [12, 13].

Recent advances in understanding the complex epigenetic alterations observed with hormone signaling, including altered histone post-translational modifications, have provided insight and opportunity for new therapeutic strategies [14–22]. Analysis of such epigenetic changes, together with changes in gene expression upon treatment with estrogens, SERM/SERD or TSECs for menopausal symptoms, provides a comprehensive and unbiased assessment of the affected regulatory networks. Modifications in the epigenetic landscape are rapid and reversible and can impact chromatin structure, as well as alter tissue-type-specific gene expression profiles. Although epigenetic changes resulting from environmental agents have been well documented, there are limited studies examining the global epigenetic changes induced by pharmaceuticals [23–25]. We therefore evaluated combinations of pharmacologic agents to identify effects of hormonal treatments with respect to their global epigenetic impact on gene expression in normal mammary epithelial and ER positive breast cancer cells.

The normal mammary gland consists of biologically distinct sets of cells that form a bi-layered, epithelial structure with outer-basal and inner-luminal cell types. We performed epigenetic profiling using two associated cell lines: MCF10A, a normal basal mammary epithelial cell line (ERα^neg^), was used as a control for the luminal MCF7 breast cancer cell line (ERα^+^). In this study, we provide evidence for selective epigenetic changes in histone modifications and the associated RNA expression profiles that result from exposure of the cells to different treatments. These include 17β-estradiol (E2), a mix of the 10 most abundant Premarin chemical equivalents (EC10), bazedoxifene (BZA) and the combination of BZA with either E2 or EC10. Epigenetic changes were examined to determine regulatory histone profiles of gene activation marks (H3K4me3, H3K4ac, and H3K27ac) and the repressive histone mark H3K27me3. This study is the first comprehensive examination of this panel of histone marks in response to currently available therapies. We identified epigenetic changes, predominantly revealed via H3K4ac, associated with both ERα bound and non-bound genomic regions as a consequence of treatment with each or combinations of the compounds. Our findings provide direct mechanistic-based evidence that BZA induces distinct changes in ERα regulatory programs to restrain the potential for breast cancer development and progression.

## Materials and methods

Experiments were conducted using MCF10A and MCF7 cell lines. Cell line authentication by short tandem repeat (STR) cell identification analysis was performed by the Vermont Integrative Genomics Resource Core (VIGR) using the Promega GenePrint® 10 System according to manufacturer’s instructions (Promega #B9510). The outcomes were compared to known ATCC fingerprints (ATCC.org) and/or the Cell Line Integrated Molecular Authentication (CLIMA) database [26] (http://bioinformatics.hsanmartino.it/clima2/).

### Cell growth conditions

MCF10A cells were grown in DMEM: F12 (Hyclone-SH30271 or Hyclone-SH302712 without phenol red), 5% (v/v) horse serum (Gibco #16050 lot #1075876) + 10ug/ml human insulin (Sigma I-1882)+ 20ng/ml recombinant hEGF (Peprotech AF-100-15) + 100ng/ml Cholera toxin (Sigma C-8052) + 0.5 ug/ml Hydrocortisone (Sigma H-0888) Pen/Strep (Life Technologies) and Glutamine (Life Technologies). MCF7 were grown in DMEM: F12 (Hyclone-SH30271 or Hyclone-SH30272 without phenol red) + 10% (v/v) FBS (Atlanta Biologicals). Prior to treatment cells were plated in serum-containing media for 24h to establish a monolayer. Medium was removed and replaced with 2% charcoal-stripped serum for 48h prior to treatment (24h) with each pharmaceutical agent. Both MCF10A and MCF7 cells were treated with 1 nM of either E2 or EC10, as this level (0.11–2.2 nM) is reported in plasma of pre-menopausal women [27, 28]. A concentration of 5nM BZA was used for this study, as a 1:5 ratio exhibited 50–60% reduction in the steroid response with either E2 or EC10 in MCF7 cells. Cells were harvested for RNA-seq or ChIP-seq analysis 24 h post-treatment as briefly described below [29].

The term EC10 defines the 10 estrogens considered the most abundant (as a percentage of the entire group of estrogens (~ 30)) in Premarin; they include 17α-dihydroequilin, 17β-dihydroequilin, equilin, 17α-estradiol, estradiol, estrone, 17α-dihydroequilenin, 17β-dihydroequilenin, equilenin, and δ-8,9-dehydroestrone. Estrone is the most abundant estrogen, representing about 50% of the entire estrogen load [30]. For in vitro use these estrogens were not conjugated. Pfizer (USA) provided the EC10 estrogens and bazedoxifene.

### RNA isolation, chromatin immunoprecipitation and library preparation

Cells were seeded at 1.0x10^6^ cells per 100mm dish and grown to 60–80% confluence prior to harvesting. Replicate treated plates were rinsed with PBS followed by mechanical scraping, pelleting and snap freezing for RNA seq. RNA was isolated using Qiagen RNeasy Plus kit (Qiagen 74134) following the manufacturer’s direction with DNase digestion, and analyzed for RNA integrity using the RNA 6000 Nano Kit with the Agilent 2100 Bioanalyzer. RNA was amplified and adaptered using the TruSeq Stranded Total RNA LT with Ribo-Zero Gold kit (Illumina RS-122-2301). Library quality was assessed by Bioanalyzer using DNA HS chip (Agilent) and quantified by Qubit (Invitrogen) and Kapa library quantification (KAPA Biosystems cat#KK4835). Libraries were single-end sequenced (SE100) on a HiSeq-1500. Base calls and sequence reads were generated by bcl2fastq software (version 1.8.4, Illumina). Three independent RNA-Seq libraries were prepared for each treatment.

For chromatin immunoprecipitation 0.8% formaldehyde was used to cross-link the protein-DNA complexes for 10 minutes at room temperature. Crosslinking was neutralized by the addition of 2.5M glycine (final concentration 0.125M) for an additional 5 minutes. Cells were washed with ice-cold PBS containing protease inhibitors (Roche-Protease Inhibitor cocktail) prior to final harvest and then flash frozen and stored at -80°C. Nuclei extraction was performed using a protocol modified from Dignam *et al*. [31]. Isolated nuclei were sonicated using a Covaris S-220 Ultrasonic Processor to obtain sheared chromatin with an average peak size of 400–500 bp. A total of 20 μg of sheared chromatin was used for ChIP immunoprecipitation with 6.7 μg of anti-H3K4me3 (Abcam, ab1012), anti-H3K27ac (Millipore, 07–360), or anti-H3K4ac (Active Motif 39382) all used at a ratio of 3:1 chromatin to antibody, or 8 μg of anti-H3K27me3 (Millipore 07–449) used at a ratio of 2.5:1 chromatin to antibody. Immunoprecipitated complexes were purified using Protein-G Dynabeads (Life Technologies 10004D lot#123085320). DNA fragments were isolated by uncross-linking the protein-DNA complex overnight at 65°C. The eluted ChIP-DNA was treated with RNase A (0.2mg/ml final concentration; Life Technologies Cat # AM2269), proteinase K treatment (Life Technologies cat # AM2548) and then phenol extracted, precipitated with ethanol, followed by resuspension in 10mM Tris. DNA was quantified using a Qubit fluorimeter (Life Technologies) prior to library preparation.

ChIP libraries were constructed using the TruSeq ChIP sample preparation kit (Illumina cat# 9235121) following the manufacturer’s instructions. Libraries were size-selected, purified and quantified prior to sequencing. Sequencing base calls were generated on a HiSeq-1500 in the VIGR Core.

### Bioinformatics analysis

#### RNA-seq

Data analysis was conducted using the Galaxy platform [32] and RStudio [33]. Quality control was performed using FastQC [34]. Reads were aligned to reference genome (hg38) using STAR [35], and quantified using HTSeq-counts [36] with Gencode annotation [37]. Differential expression analysis was performed with DESeq2 [38]. For differential gene expression analyses, the cutoff for significant fold change was >2, adjusted p-value <0.05.

#### ChIP-seq

Fastq conversion and demultiplexing were done using bcl2fastq (Illumina, v1.8.4), evaluated (Fastqc), processed to remove low quality reads, and trimmed (FastX toolkit). Reads were mapped to the human genome (hg38) using STAR aligner (version 2.4) with splicing disabled (—alignIntronMax 1) [35]. Enriched regions (narrowPeak calls) for each replicate were generated using MACS2 [39] and replicates were then evaluated using bamCorrelate and IDR. After pooling replicates, MACS2 was used on H3K4me3 to call narPeak at high stringency (p-value < 10e-5) and SICER V1.1 [40] was used to identify broad H3K27me3 enrichment domains. SICER results were filtered by consensus using a range of window-gap combinations (windows 200, 300, 375, and 400; gaps 600, 1200, 1125, and 2400 respectively). Data were deposited in the NCBI Gene Expression Omnibus (GEO) (GSE102472). ERα data were obtained from NCBI GEO GSE40129 [41].

Reactome pathway analysis of BZA effect gene groups was performed using the hypeR R package with hypergeometric test [42]. KEGG pathway analysis of genes associated with drug-responsive H3K4ac changes was performed using the enrichKEGG function from the clusterProfileR package [43]. Differential ChIP-seq analysis was performed using csaw (1.20.0) [44, 45] and was analyzed according to the standard workflow described in the "csaw User Guide" on bioconductor for all drug treatment combinations per cell line and histone modification. Window counts were generated using a fragment extension of 160, spacing of 50, and width of 50 with duplicates ignored (picard 1.56.0; MarkDuplicates). The ENCODE hg38 genomic region blacklist (version 1) was used to avoid problematic regions. An FDR filtering threshold of 0.05 was applied and all resulting regions were compared to establish a set of 148 sites where any histone modification change is detected.

### Validation by reverse transcriptase quantitative PCR (RT-qPCR)

Biological replicates were performed using total RNA purified using RNeasy column purification (Qiagen); samples were treated with DNase I and reversed transcribed with Multiscribe reverse transcriptase (Applied Biosystems). Real-time PCR was performed with SYBR Green (BioRad) on the QuantStudio7 Pro Real-Time PCR System (Applied Biosystems). Samples were normalized using primers for HMBS: CTGCAAGCGGGAAAACCCT (F), CTCCAGATGCGGGAACTTTCT (R); TBP: GGAGAGTTCTGGGATTGATC (F), CTTATCCTCATGATTACCGCAG (R); HPRT: TGCTGACCTGCTGGATTACA (F), TCCCCTGTTGACTGGTCATT (R); and ACTB: AGCACAGAGCCTCGCCTTT (F), CGGCGATATCATCATCCAT (R). Specific genes evaluated using primer sequences (5’-3’) are GREB1: GGTCTGCCTTGCATCCTGATCT (F), TCCTGCTCCAAGGCTGTTCTCA (R) and TFF1: CCAGTGTGCAAATAAGGGCTGC (F), AGGCAGATCCCTGCAGAAGTGT (R). The relative quantitation was performed using the ΔΔCt method. The log2FC values were calculated from the ratios of the ΔΔCt values for MCF7 cells (untreated) vs E2, EC10, BZA, E2/BZA, and EC10/BZA.

### Statistical analysis

For ChIP-seq analysis, 3 independent replicates were used to generate peak calls (MACS2) and to calculate average log2 fold change (FC) relative to the untreated control for each cell line. MACS2 employs a stringent Poisson distribution model to capture both the mean and the variance of the distribution over peak areas. Significant differences in relative changes in fold enrichment were evaluated by pair-wise t-test and false discovery rate calculated by Benjamini–Hochberg procedure and expressed as mean normalized fold-enrichment values. For RNA-seq experiments, at least 3 independent replicates were used to evaluate RNA expression. Statistically significant differences between the means was determined by DESeq2 using a modified Wald test and expressed as mean log2-fold changes.

## Results

### Bazedoxifene treatment tempers steroid-responsive changes in gene expression

**Fig 1:**
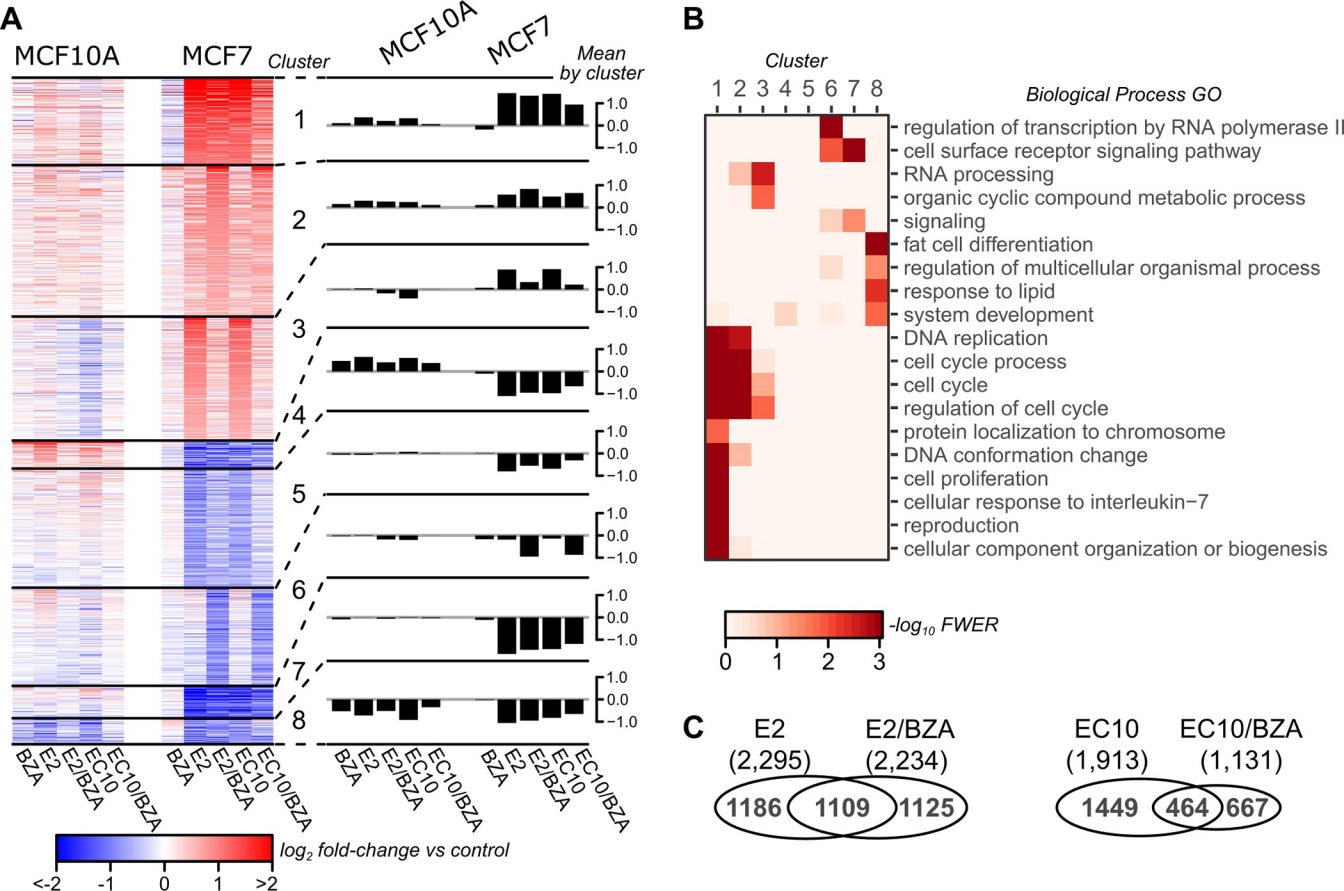
Gene expression changes with SERM/SERD treatments (bazedoxifene–BZA) in MCF10A and MCF7 cell lines, as evaluated by RNA-seq. A) Clustering of 3,820 differentially expressed genes. Values are average log2 fold change (FC) of normalized counts in 3 independent replicates relative to the untreated control for each cell line. Eight clusters were calculated using k-means. Data for individual genes are presented in the left panel heatmap, and mean values (log2 fold-change) in each cluster are presented in the right panel barplots. B) Biological process gene ontology (GO) enrichment per cluster. Terms shown are the least redundant terms as determined by REViGO. C) Venn diagram comparing differentially expressed genes in MCF7 between E2 vs E2/BZA (left) and EC10 versus EC10/BZA (right); each of the 4 groups are relative to control.

We studied the gene expression profiles (by RNA-seq) of the estrogen receptor agonists EC10 and 17β-estradiol (E2) alone, or combined treatment with the ERα antagonist bazedoxifene (BZA) in the human mammary cell lines MCF10A^ER-^ and MCF7^ER+^. Cells were hormone starved for 48h and then treated with each agent for 24h prior to RNA collection. Principal Component Analysis was performed to assess the global gene expression patterns across each treatment group using 3 replicates per treatment for both MCF7 and MCF10A. In MCF10A, we do not detect clustering of samples for any of the treatments (S1 Fig, left). However, MCF7 samples treated with the estrogen formulations E2 and EC10 cluster together (S1 Fig, right). Similarly, BZA and control treatments (vehicle) cluster together, whereas the combinations of BZA with E2 or EC10 cluster separately, indicating each had different effects. We performed differential expression analysis among each of the 5 treatment groups for each cell line MCF10A and MCF7, followed by k-means clustering of fold-change values relative to untreated controls (Fig 1A). As expected, there are minimal changes for any of the treatments in the MCF10A cell line. In contrast, ER-positive MCF7 cells exhibit robust gene expression changes in response to E2 and EC10. BZA alone resulted in minimal changes in gene expression, consistent with a previous report. However, combinations of BZA with either E2 or EC10 had substantial effects on gene expression, and strongly reduced the expression of genes in clusters 3 and 6 (Fig 1A). In cluster 3, the expression of genes up-regulated by E2 and EC10 are reduced to near control levels when BZA is present. In cluster 6, the expression of genes that are non-responsive to E2 and EC10 are down-regulated when BZA is present. The responsiveness observed for combinations of BZA with E2 or EC10 is consistent in direction (up or down) but not in magnitude (high or low).This variation with combinations of E2 or EC10 with BZA indicates that the expression levels of subsets of genes are more sensitive to the combined therapy of E2/BZA or EC10/BZA. The observed patterns of estrogen responsiveness are consistent with known ERα genomic activity in breast cancer cells, and the E2 or EC10 (agonist) up-regulated clusters 1–3 are enriched for cell proliferation and cell cycle gene ontology categories (Fig 1B). The clusters where combination with BZA has unique effects include genes enriched in cell cycle and RNA processing pathways (Cluster 3) or RNA polymerase II transcription and cell surface receptor signaling (Cluster 6). These results demonstrate that BZA in combination with E2 or EC10 attenuates the expression of distinct clusters of estrogen-responsive genes.

To determine distinctive expression effects elicited by the different drug combinations, pairwise comparisons were performed between treatment groups in MCF7 cells. Relative to control, the agonists E2 and EC10 altered the expression of many genes that are comparable between the two agonist treatments (2,295 and 1,913 differentially expressed genes, respectively) (Fig 1C). However, E2 combined with BZA resulted in a greater number of differentially expressed genes than when EC10 was combined with BZA. E2/BZA changes the expression of 2,234 total genes versus only 1,131 for EC10/BZA. Thus, BZA has a selective effect on ER agonist-induced gene regulation.

### BZA selective and combinatorial regulation of ER agonist genes

**Fig 2:**
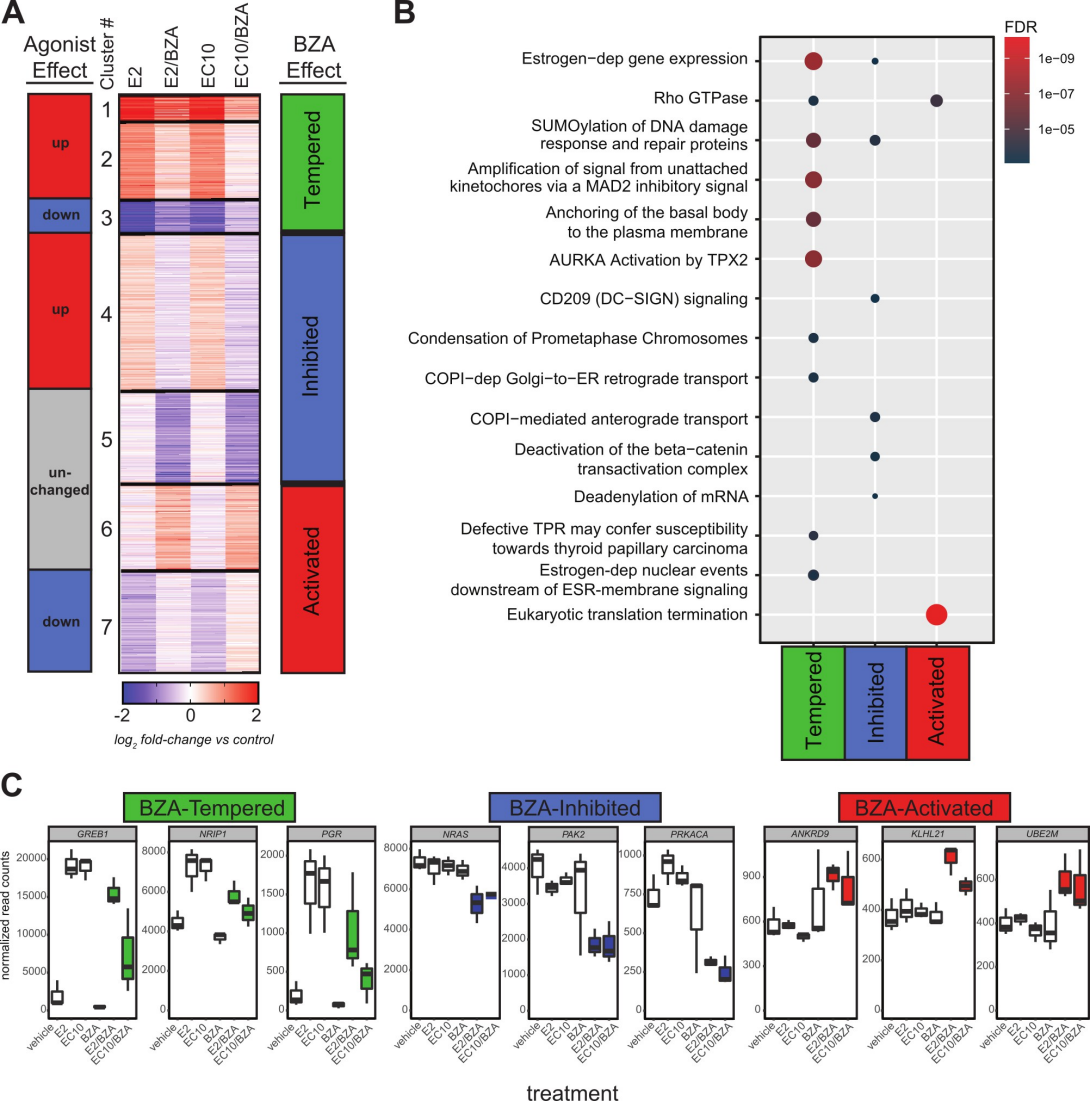
BZA alters steroid responsiveness in the MCF7 cell line. A) Clustering analysis of 2,332 genes comparing BZA-induced changes in E2 or EC10 responses. There are seven distinct clusters that group into different agonist effect groups (E2 or EC10) or into three different BZA-effect groups (Tempered, Inhibited, Activated) based on the effect of BZA on agonist responses. B) Reactome pathway enrichment analysis of BZA-effect groups from Fig 2A. The top enriched Reactome gene sets are shown in dot plot, where each dot is an enriched gene set, the color represents the significance, and the size signifies the gene set size. C) Normalized RNA-seq expression values of selected genes from each BZA-effect group (Tempered, Inhibited, Activated). Data shown are from MCF7 RNA-seq DESeq2-normalized read counts from each treatment group (n = 3 replicates per group). The E2/BZA and EC10/BZA treatments are color-coded according to the BZA-effect groups from Fig 2A.

The expression responses elicited by individual agonist and BZA combination treatments were further investigated by clustering all MCF7-responsive genes (2,332 total genes) (Fig 2A). Responsive genes were grouped into 7 distinct clusters based on patterns of expression relative to vehicle control. The effects of E2 and EC10 on genes could be classified into separate agonist-effect groups (up, down or unchanged genes), as well as into distinctive ‘BZA effect’ groups (tempered, inhibited or activated ‘BZA effect’ genes). In particular, BZA reduced or ‘tempered’ the ER agonist-induced responses for the estrogen-activated genes in clusters 1 and 2 and for the estrogen-repressed genes in cluster 3. Clustering analysis also revealed that combination of BZA with E2 or EC10 altered the expression of non-estrogen responsive genes in the agonist-unchanged gene groups (the ‘inhibited’ cluster 5 and the ‘activated’ cluster 6) (Fig 2A; S1 Table).

The effects of BZA on biological pathways were determined by Reactome pathway enrichment analysis. The BZA-tempered group (clusters 1, 2 and 3) is comprised of agonist-responsive genes that are strongly enriched in estrogen-dependent gene expression pathways, as well as several other pathways. Included are SUMOylation of DNA damage and repair proteins, and those involved with spindle assembly during mitosis, such as amplification of signal from unattached kinetochores via a MAD2 inhibitory signal, anchoring of the basal body to the plasma membrane and AURKA Activation by TPX2 (Fig 2B; S2 Table). BZA-tempered genes also include a number of known estrogen receptor target genes such as *GREB1*, *NRIP1*, and *PGR* (Fig 2C). Expression of GREB1 as a BZA-tempered gene was confirmed by qPCR analysis (S2 Fig); TFF1, the trefoil gene, is another gene that is regulated upon BZA treatment. Both *GREB1* and *TFF1* are well characterized estrogen-responsive ERα target genes. The BZA-inhibited group (clusters 4 and 5) is comprised of genes that share pathways with the BZA-tempered groups, as well as those enriched in CD209 (DC-SIGN) signaling and deactivation of the beta−catenin trans-activating complex pathways. BZA-inhibited genes include *NRAS*, *PAK2*, and *PRKACA*. Lower expression is observed with BZA-combination treatments than with either E2 or EC10 agonists alone (Fig 2C). The BZA-activated genes in clusters 6 and 7 are enriched in Rho GTPase and eukaryotic translation termination pathways, and include the genes *ANKRD9*, *KLHL21* and *UBE2M* (Fig 2C). Overall, these results show that BZA in combination with ERα agonists alters both estrogen-dependent and estrogen-independent gene regulation.

### MCF10A and MCF7 exhibit distinctive distributions of chromatin states

**Fig 3:**
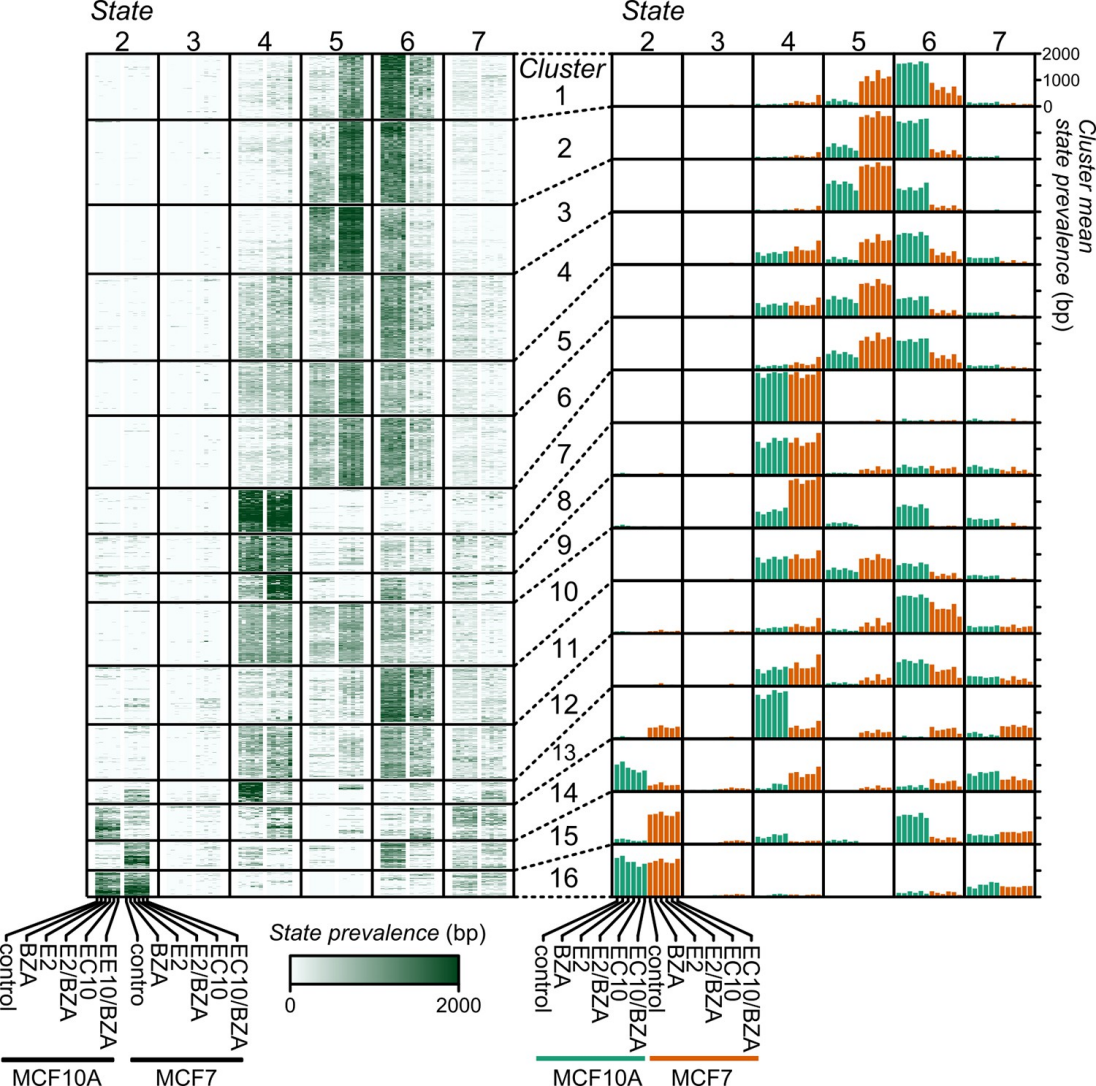
Chromatin state dynamics near RefSeq gene Transcription Start Sites (TSS) in MCF10A and MCF7 cells treated with bazedoxifene. The number of base pairs assigned to each state is within a 2kb window centered on 18,614 TSSs clustered and presented in heatmaps (left) or aggregated barplots (right). State 1 is omitted due to its extreme rarity in order to improve clarity. Minor columns represent the prevalence of the state in each cell line and treatment combination as indicated at the bottom, repeating in each major column (left panel). For the right panel, the clusters are summarized by mean value and the columns are arranged as in the left panel. Chromatin states: 2 –repression; 3 –mixed signal; 4 –low signal; 5 –active; 6 –poised; 7 –low signal.

To address how the estrogen compounds in combination with BZA treatment impact the chromatin landscape, we performed ChIP-seq analysis for both MCF10A and MCF7 cells, using a panel of regulatory histone marks for each treatment group. Duplicate ChIP-seq assays for each of the 4 different regulatory histone modifications were carried out, including histone H3 lysine 4 tri-methylation or acetylation (H3K4me3 or H3K4ac), both associated with active promoters, and histone H3 lysine 27 acetylation or tri-methylation (H3K27ac or H3K27me3) associated with active regulatory regions or polycomb-repressed regions, respectively. Using these data, we first evaluated the chromatin state landscape (ChromHMM) of transcription start sites (TSSs) across all protein-coding genes (Fig 3 and S3 Fig). For example, for clusters 1–6 there is a shift from state 6 (poised) in MCF10A to state 5 (active) in MCF7. Further, there is a shift in cluster 14 from state 2 (repression) in MCF10A to state 4 (low signal) in MCF7, and a shift in cluster 15 from state 6 (poised) in MCF10A to state 2 (repression) in MCF7 (all genes annotated to each cluster are listed in S3 Table). Thus, the chromatin state distinctions between the MCF10A and MCF7 cell lines are dominant over the effects of the different treatment groups.

### Steroid treatments impact distinct regulatory elements

**Fig 4:**
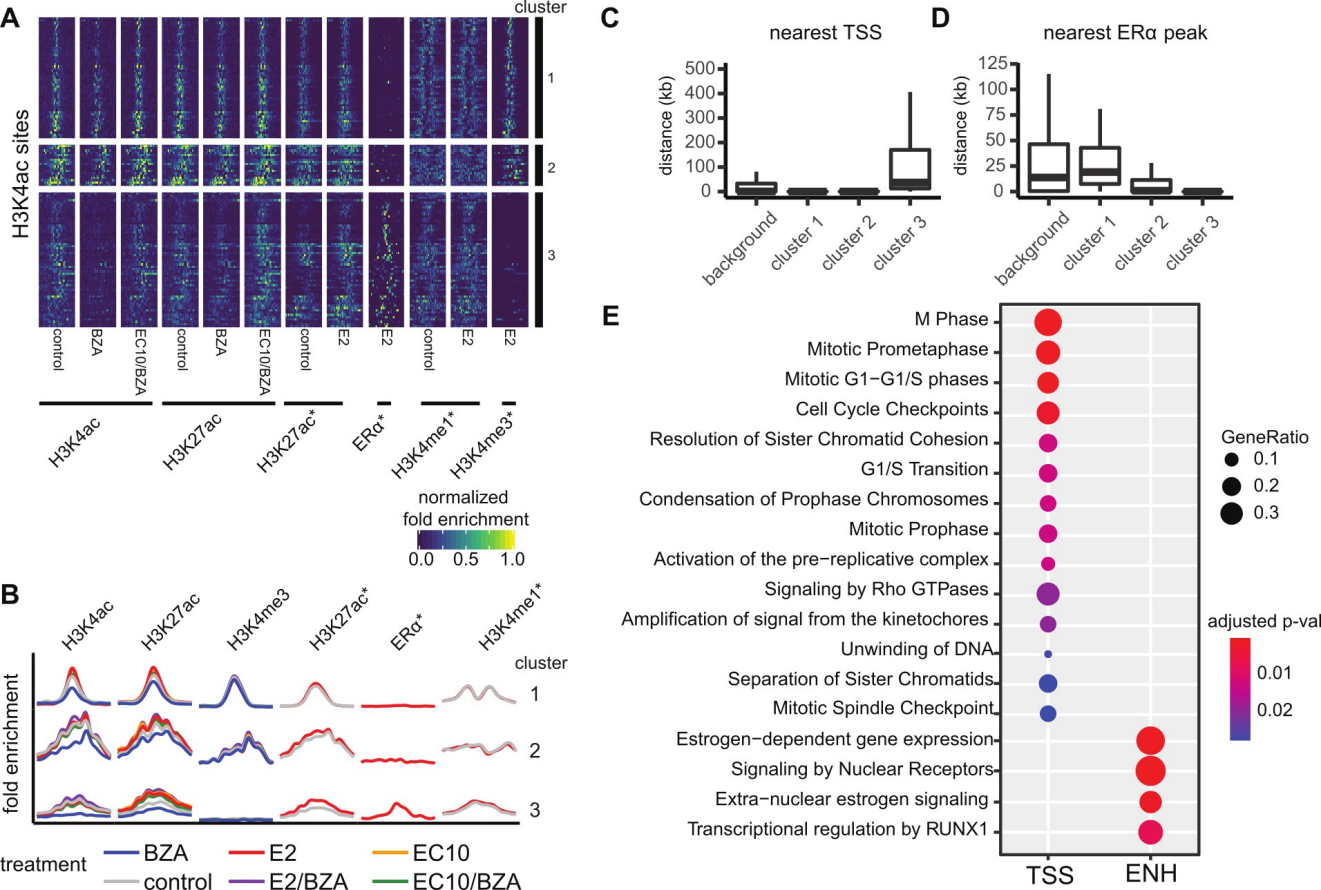
H3K4ac is regulated in response to bazedoxifene treatment in MCF7 cells. Clustering of 130 differentially enriched H3K4ac marked regions reveals two distinct epigenetic signatures. A) Heatmap of enrichment profiles across genomic regions within 4kb of H3K4ac sites clustered using k-means (k = 3). B) Aggregated mean profiles of clusters identified in A; the x-axis corresponds to the mean normalized fold-enrichment values. C) Distance by cluster from each site to the nearest transcript start site (TSS) of a protein-coding gene. D) Distance by cluster from each site to the nearest ERα peak. E) KEGG enrichment analysis of pathways associated with genes nearby cluster 1 and 2 promoter proximal regions (TSS) or cluster 3 enhancer (ENH) regions.

Because the chromatin state patterns are driven by cell line differences and reveal limited information about global epigenetic consequences of the drug treatments, we directly addressed the differential epigenetic profiles of genomic regions that are responsive to our drug panel. We conducted differential enrichment analysis of ChIP-seq data between each treatment and control. In MCF10A cells, no significantly altered regions were detected across any of the treatment groups compared to controls. However, in MCF7 cells 130 genomic regions were significantly altered for each of the 3 active marks, H3K4me3, H3K27ac, and H3K4ac. No differentially enriched regions were found for the repressive H3K27me3 mark in MCF7 across treatments. We therefore limited further analysis to the active marks in MCF7, focusing on the BZA and BZA/EC10 treatments versus control (Fig 4A). The H3K4ac mark is the most sensitive to the different treatments compared to the other histone modifications (S4 Fig), and the set of H3K4ac treatment-changed regions represents most of the detected epigenetic alterations in MCF7. Our earlier studies of H3K4ac have shown this mark to be more dynamic than H3K4me3 in response to estrogen signaling in breast epithelial and ER-positive breast cancer cell lines [46].

Using H3K4ac sites, we identified clusters of genomic regions that displayed different enrichment patterns with treatments (Fig 4A and 4B) that are consistent with both gene promoter regions (clusters 1 and 2) and distal gene enhancer regions (cluster 3). Comparison of our data with ERα ChIP-seq data [41] identified promoter clusters that are defined by the presence of H3K4me3 and the absence of ERα (Fig 4B and 4C), whereas the enhancer cluster lacks H3K4me3 but is enriched with ERα (Fig 4B and 4D). Strikingly, BZA treatment resulted in a complete loss of H3K4ac at enhancer sites, while the response is more muted at gene promoter regions. The H3K4ac responsive gene promoter regions not bound by ERα (clusters 1 and 2) correspond to genes associated with cell cycle regulation, DNA metabolic processes and mitotic checkpoints (Fig 4E; TSS). Genes that annotate to enhancer regions (cluster 3) are enriched with signaling pathways of validated estrogen networks and include several estrogen-related pathways in addition to transcriptional regulation by RUNX1, a pathway linked to breast cancer and tumor suppression [47–51]. Of significance, a role for RUNX1 in mediating ERα tethering versus direct genomic recruitment to estrogen response elements has been reported [52]. We have therefore detected two modes in which BZA can impact global patterns of histone modifications, one being promoters that are not directly associated with ERα but are highly related to cell cycle and proliferation, and the other being enhancers that are directly associated with ERα and known estrogen gene regulatory networks.

### Drug-induced H3K4ac alterations are associated with gene expression

**Fig 5:**
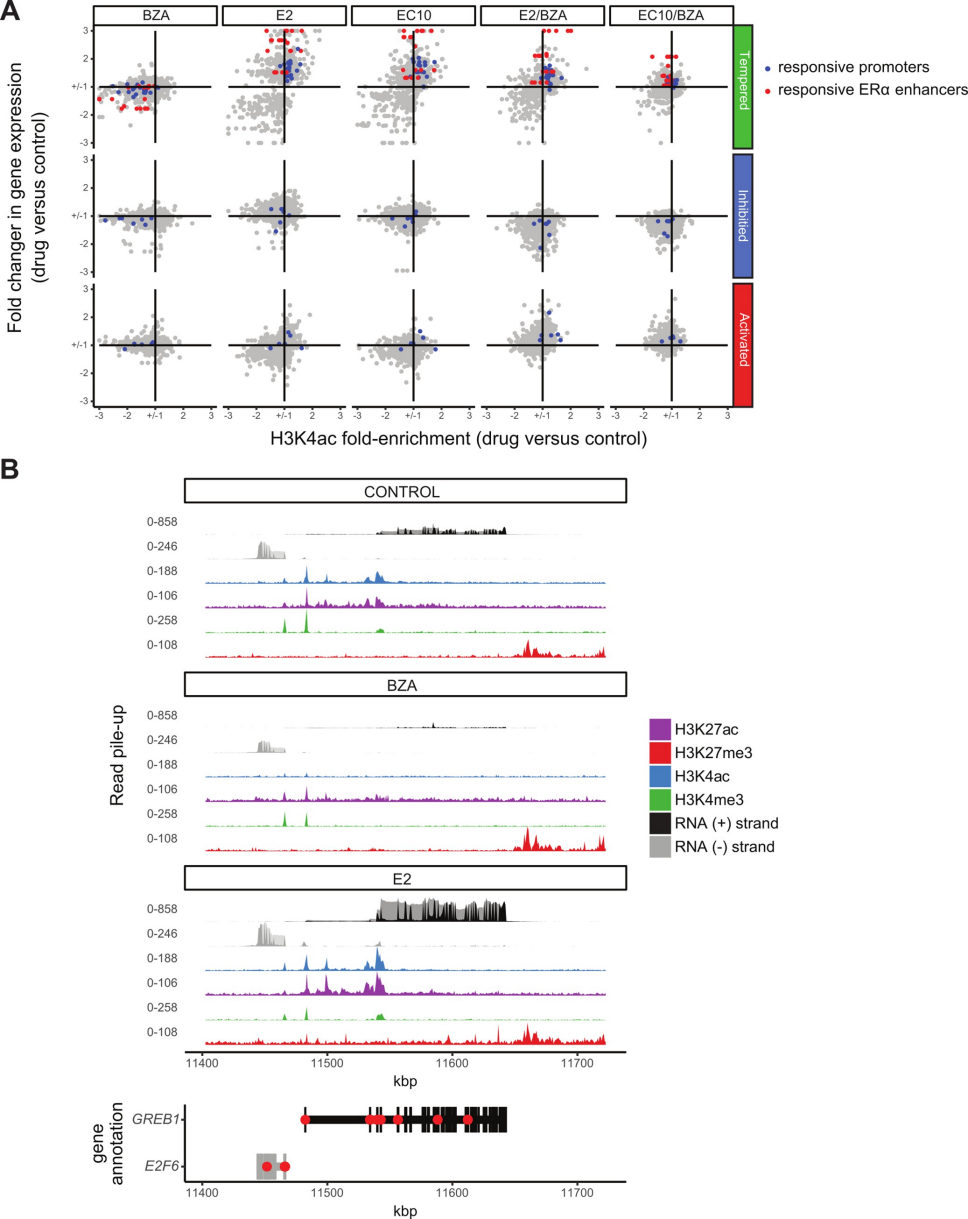
Changes in H3K4ac correspond to expression changes for nearby genes. A) Scatterplots showing the fold-change in gene expression versus the fold-enrichment change in H3K4ac signal for each drug treatment versus control, respectively. The two types of significantly differentially enriched H3K4ac regions are shown separately for genes that belong to each BZA-effect group (Tempered, Inhibited and Activated). The color of the dots is based on clustering from Fig 4 and corresponds to the genes of non-ERα bound promoters (blue points) and ERα bound enhancers (red points). Fold enrichment changes are capped at 3 for each comparison. B) Effects of BZA and E2 on GREB1 gene expression and histone modifications relative to Control.

We next examined the relationships between drug-induced H3K4 acetylation changes and corresponding gene expression responses. Because BZA in combination with ERα agonists alters the expression of both estrogen-dependent and estrogen-independent genes, we examined these groups separately (Tempered, Inhibited and Activated BZA-effect gene groups; Fig 2A). BZA-tempered genes show an overall reduction in agonist-induced regulatory changes, both in terms of the degree in fold-change expression and in fold-change H3K4ac signal (Fig 5A). This observed reduction occurs similarly at the BZA-responsive promoters and ERα enhancers (blue and red dots, respectively). In contrast to the BZA sensitivity at tempered genes, BZA-inhibited and activated genes show reciprocal changes in gene expression (increased in BZA-activated and decreased in BZA-inhibited), with minimal changes in H3K4ac enrichment, entirely at the TSS/promoter regions of non-ERα bound genes. Treatment with BZA alone shows minimal RNA expression changes, but a strong overall reduction in H3K4ac enrichment; these epigenetic changes are predictive of expression changes when BZA is combined with E2 or EC10. These results indicate that BZA treatment reduces ERα activity at gene regulatory elements nearby target genes.

Two of the most highly regulated genes upon BZA treatment are *GREB1* and *TFF1*, both well characterized as estrogen-responsive ERα target genes. Treatment with E2 (or EC10) increases H3K4ac at the *GREB1* and *TFF1* enhancers (GREB1 shown in Fig 5B) and at the *GREB1* promoter with a dramatic increase in RNA expression (>7-fold). In contrast, treatment with BZA shows a more pronounced loss in H3K4ac at *GREB1* enhancer sites (4-fold change) compared to the *GREB1* promoter (2-fold change, when compared to the vehicle control). The increased H3K4ac enrichment and expression change at *GREB1* are expected, as this region is a highly E2-responsive ERα target gene [11, 53, 54]. Overall, these results indicate H3K4ac is a dynamic histone modification that is more sensitive to BZA treatment than the other histone modifications examined and impacts both ERα-regulated and non-ERα genes.

## Discussion

Estrogens exert biological control via binding to estrogen receptors, which in turn regulate transcriptional events and downstream gene expression programs in a tissue-specific manner. These actions can be mediated by direct binding of estrogen receptor complexes to specific regulatory elements, or through non-genomic mechanisms that do not involve direct binding to DNA [15, 19, 52, 55–57]. Our findings in this study suggest that bazedoxifene in conjunction with estrogens produces an augmented profile of estrogen-dependent proliferative and stimulatory responses in breast cells. The profile with BZA alone is similar to that observed in the normal ERα^neg^ MCF10A cell line. In contrast, estrogen complexes (estrogens in combination with BZA) minimize the expression of estrogen-responsive genes normally induced by estradiol or EC10 in ERα^pos^ MCF7 cells.

Using an integrated analysis, we evaluated the impact of combination therapies on gene expression and histone modifications (H3K4me3, H3K4ac, H3K27ac, H3K27me3) in estrogen-responsive (MCF7) and non-responsive (MCF10A) cell lines. We observed minimal changes in both epigenetic marks and gene expression in the breast epithelial MCF10A cell line. These findings reveal that in ERα^neg^ MCF10A cells there are nominal off-target effects attributable to the estrogen and BZA compounds we examined. In contrast to the MCF10A mammary epithelial cells, ERα^+^ MCF7 breast cancer cells demonstrate an increase in activating histone marks H3K4ac, H3K4me3 and H3K27ac upon treatment with either steroid compound (E2 or EC10). This is consistent with the known effects of estrogen compounds [46, 58–60]. In particular, we find that the enrichment of H3K4ac was impacted at the promoters of proliferation and cell cycle gene pathways. Accordingly, we observed that increased histone H3K4ac and RNA expression changes were mitigated by the addition of BZA, which shifted these parameters towards levels present in untreated cells. Treatment with BZA alone showed negligible changes in global H3K4ac and RNA transcript levels, with the exception of a small subset of genes—most notably *GREB1*, and its associated enhancer.

Our findings highlight an important role for H3K4ac in the measure of estrogen responsiveness [46]; compared to the other histone marks analyzed, H3K4ac is the best indicator of epigenetic changes in response to drug treatments. H3K4ac has been shown to be present at both promoters and enhancers [61, 62]. When we interrogate all H3K4ac changes with respect to ERα occupancy [41], we find that enhancers bound by ERα are particularly sensitive to E2 and EC10 treatment. However, upon addition of BZA to E2 or EC10 treatment, H3K4ac decreases significantly at both ERα-bound and non-bound regions. ERα-bound regions demonstrate a more dramatic H3K4ac response to all BZA treatments, with almost a complete loss of H3K4ac with BZA alone (Fig 4). The promoter-distal regions marked by H3K4ac and H3K27ac and bound by ERα are reminiscent of estrogen-responsive genomic enhancers. Indeed, one of the most highly changed gene enhancer regions in our study is a well-known estrogen response element (ERE) upstream of *GREB1* [54]. Genes exhibiting responses to ERα are enriched within canonical ER response pathways, including those involved with cell cycle regulation and RNA processing. While these changes were expected for ER agonists, the potent mitigation by BZA of the ER-mediated epigenetic changes in H3K4ac is a novel observation.

The steroids EC10 and E2 stimulated widespread regulatory changes in MCF7 cells, and the effect of these treatments was strikingly reduced by the ER antagonist bazedoxifene (BZA). In particular, BZA co-treatment restricted the specific changes in estrogen-induced gene expression. We find through clustering and pathway analysis (Fig 2) that BZA co-treatments with ER agonists E2 or EC10 have the potential to augment hormone responsive gene regulation. Collectively, our data provide a valuable resource for understanding epigenetic signatures associated with estrogen pathways, as well as a comprehensive picture of the changes in acetylation at histones H3K4 and H3K27 that reflect the altered functional organization of the genome in response to BZA. Estrogen-mediated gene regulation has been functionally linked to cancer initiation and progression. There is potential to address the capabilities of BZA to block proliferation in metastatic, hormone refractory breast cancer cell lines, such as MDA-MB-231, or in ER-positive metastatic breast cancer cell lines, such as BT-474. Our findings are consistent with BZA-mediated epigenetic control that sustains physiological regulation of proliferation and phenotype by preventing estrogen-compromised gene expression.

## Supporting information

S1 Fig Gene expression changes with drug treatment relative to control. Principal Component Analysis (PCA) of global gene expression profiles for each drug treatment using DESeq2 rlog-normalized RNA-seq data for MCF10A (left) and MCF7 (right).
(TIF)

S2 Fig Validation of RNA-seq data analysis using RT-qPCR. Relative mRNA expression levels for (A) GREB1 and (B) TFF1 were evaluated in biological replicate sample sets generated independently from RNA used for RNA-seq libraries and measured by RT-qPCR.
(TIF)

S3 Fig ChromHMM states capture cell line differences. A) The top panel describes emission probability for 4 marks for the 7 state model, the middle panel overlaps states with different genomic feature types in MCF10A, and the bottom panel shows these feature types for MCF7. MCF10A shows higher occurrence compared to MCF7 of state 6 at promoter-associated features (CpG Island and RefSeqTSS 2kb, most notably). **B**) Both *GATA3* and *CDKN2C* promoters exhibit a shift from predominantly state 6 with some state 5 in MCF10A to almost entirely state 5 in MCF7. Chromatin states: 1 –weak repression; 2 –repression; 3 –mixed signal; 4 –low signal; 5 –active; 6 –poised; 7 –low signal.
(TIF)

S4 Fig Differential H3K4ac demonstrates most significant drug-induced changes in histone modifications in MCF7 cells. Each treatment pair is composed of a direct differential binding analysis (see methods) across both MCF10A and MCF7 datasets. In the binary heatmap, the red and gray bars indicate significant differentially enriched histone modifications (FDR < 0.05) across each treatment pair.
(TIF)

S1 Table Lists of MCF7 2,332 hormone responsive genes grouped into clusters 1–7 or by BZA-effect groups (Fig 2A). (XLS)

S2 Table Enriched Reactome pathways of BZA-effect gene groups from Fig 2A. (XLS)

S3 Table Gene names that correspond to the TSSs from the ChromHMM clusters from Fig 3. (CSV)
