# Supplementary figures and images for: Epigenetic and transcriptome responsiveness to ER modulation by tissue selective estrogen complexes in breast epithelial and breast cancer cells
Source: PLoS One. 2022 Jul 21;17(7):e0271725. doi: 10.1371/journal.pone.0271725 (PMC9302754; doi:10.1371/journal.pone.0271725)

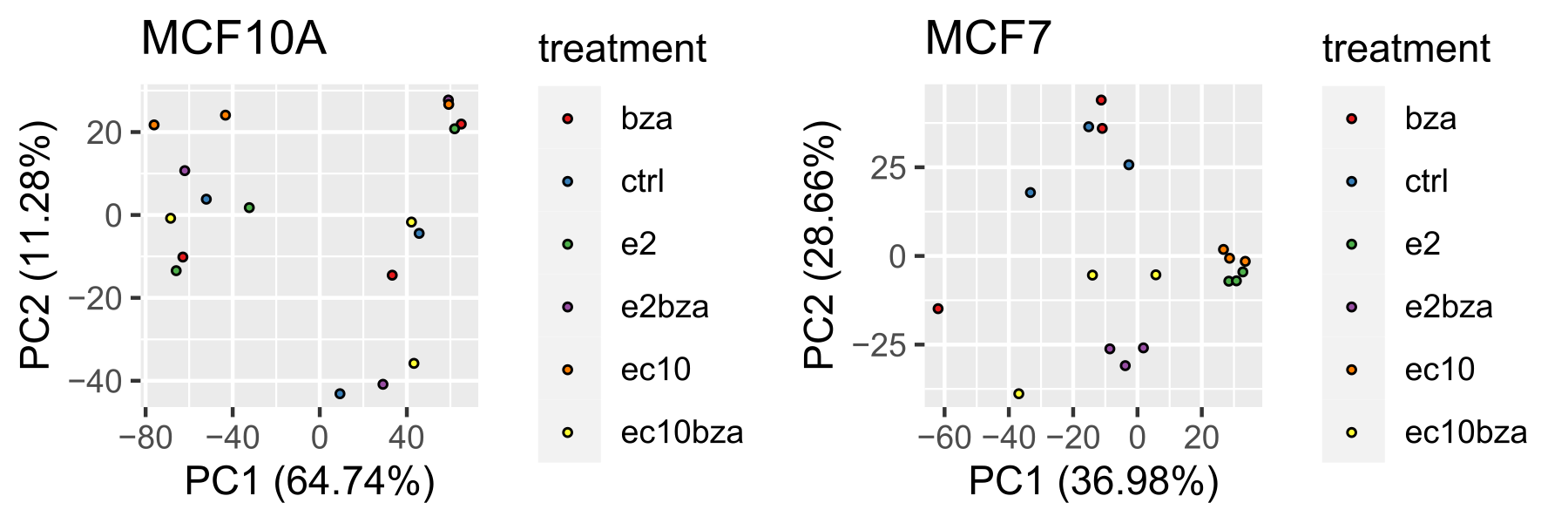

Supplement: S1 Fig — Principal Component Analysis (PCA) of global gene expression profiles for each drug treatment using DESeq2 rlog-normalized RNA-seq data for MCF10A (left) and MCF7 (right). (TIF) [file pone.0271725.s001.tif]

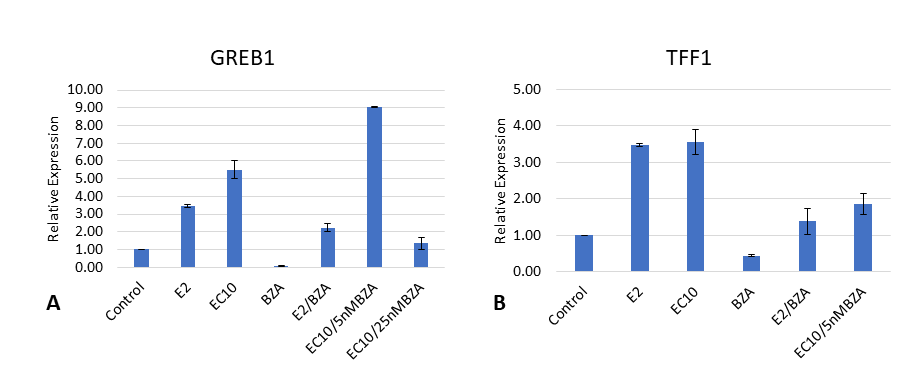

Supplement: S2 Fig — Relative mRNA expression levels for (A) GREB1 and (B) TFF1 were evaluated in biological replicate sample sets generated independently from RNA used for RNA-seq libraries and measured by RT-qPCR. (TIF) [file pone.0271725.s002.tif]

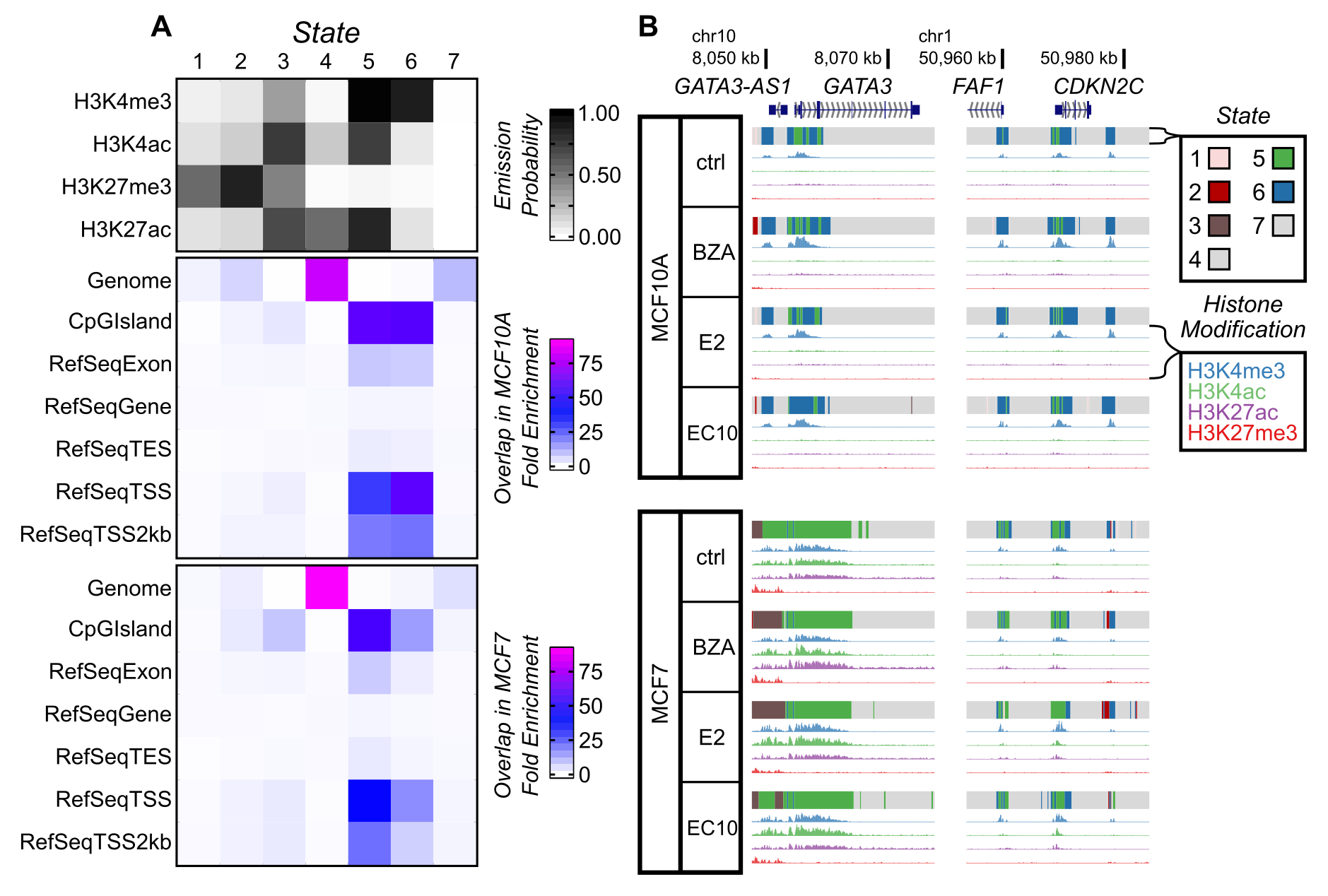

Supplement: S3 Fig — A) The top panel describes emission probability for 4 marks for the 7 state model, the middle panel overlaps states with different genomic feature types in MCF10A, and the bottom panel shows these feature types for MCF7. MCF10A shows higher occurrence compared to MCF7 of state 6 at promoter-associated features (CpG Island and RefSeqTSS 2kb, most notably). B) Both GATA3 and CDKN2C promoters exhibit a shift from predominantly state 6 with some state 5 in MCF10A to almost entirely state 5 in MCF7. Chromatin states: 1 –weak repression; 2 –repression; 3 –mixed signal; 4 –low signal; 5 –active; 6 –poised; 7 –low signal. (TIF) [file pone.0271725.s003.tif]

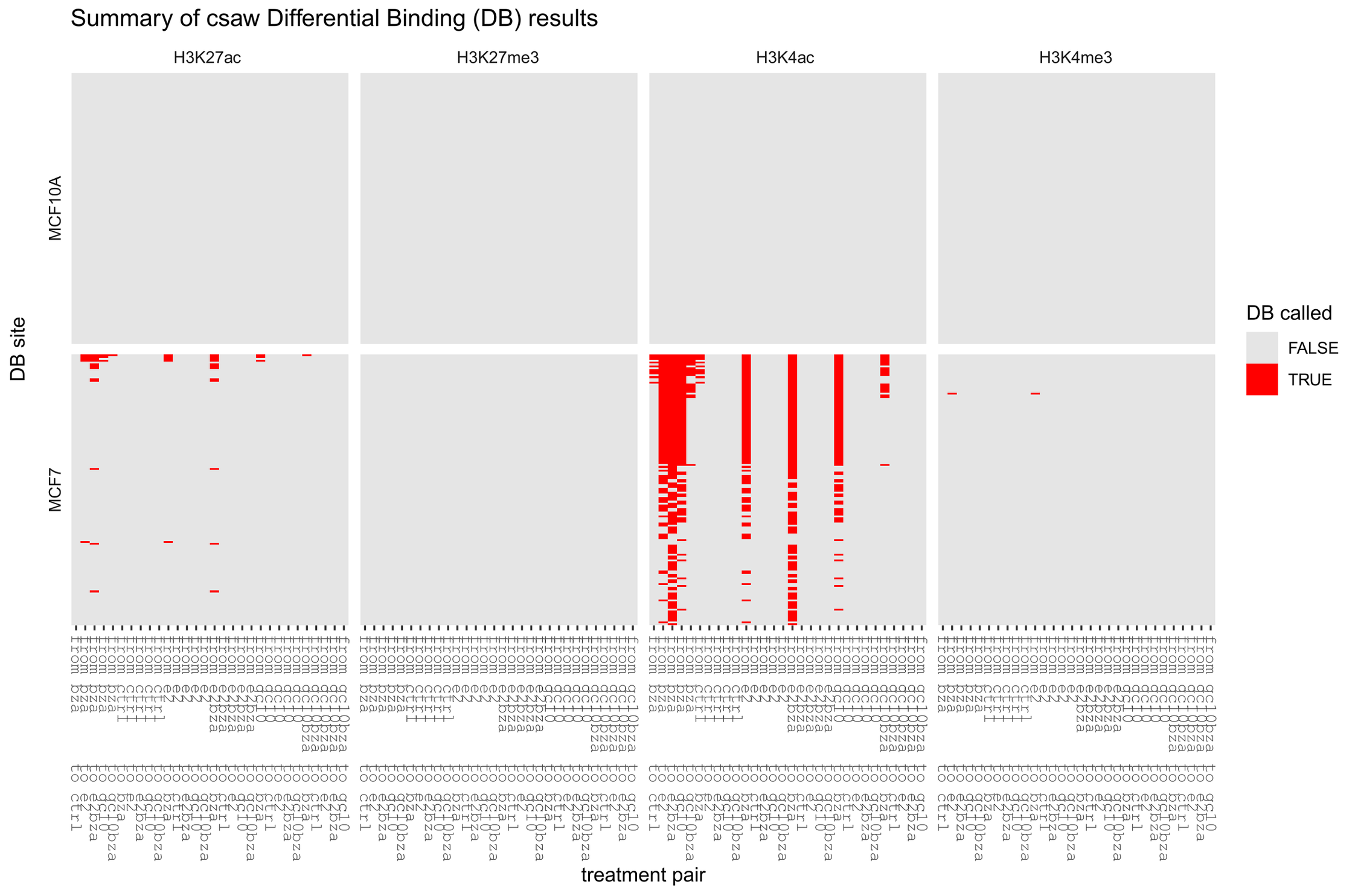

Supplement: S4 Fig — Each treatment pair is composed of a direct differential binding analysis (see methods) across both MCF10A and MCF7 datasets. In the binary heatmap, the red and gray bars indicate significant differentially enriched histone modifications (FDR < 0.05) across each treatment pair. (TIF) [file pone.0271725.s004.tif]
